# Case Report: J-Shaped External Fixator for Treatment of Mayo Type II Olecranon Fractures – A Novel Surgical Technique and Report of Clinical Applications

**DOI:** 10.3389/fsurg.2022.855600

**Published:** 2022-06-15

**Authors:** Yue Tian, Xin Ge, Jiyang Zou, Fenglei Song, John Chun tien chui wan Cheong, Changqing Ge, Weiguo Zhang, Jie Li, Kang Tian

**Affiliations:** ^1^ Department of Orthopedics, No. 2 Hospital of Baoding, Chengde Medical University, Baoding, China; ^2^ Department of Bone and Joint, First Affiliated Hospital, Dalian Medical University, Dalian, China; ^3^ Emergency Center, First Central Hospital of Baoding, Baoding, China

**Keywords:** external fixation, elbow, olecranon fractures, case series, fracture

## Abstract

**Purpose:**

We designed a J-shaped external fixator (J-EF) to provide a minimally invasive, one-step surgical method for olecranon fractures. The aim of this study is to retrospectively review the method and the outcomes in 14 patients treated with J-EF fixation.

**Methods:**

Biomechanical comparative study was performed to test the tensile properties of the J-EF using a universal testing machine. Between January 2002 and December 2005, 14 patients (age range: 25–67 years) with Mayo type II olecranon fractures were treated using the external fixation technique. Follow-up was done by standard measures (radiography, range of motion, and complications monitoring) and patient-reported outcomes (Mayo Elbow Performance Score [MEPS] and Disabilities of the Arm, Shoulder, and Hand [DASH] scores) 6 months after surgery. Eight of the patients were reviewed 15 years after the surgery.

**Results:**

Results from biomechanical studies indicate the non-inferiority of J-EF to tension-band wiring (TBW) in tensile properties. At the time of release, the mean elbow flexion arc was 132.5° and the mean forearm rotation arc was 173.6°. The mean DASH score was 14.1 points, and the mean MEPS was 93.9 points. Operative time and intraoperative blood loss were decreased by 41.3% and 64.6%, respectively, in J-EF patients than those in a comparable group treated by TBW. All eight patients are still alive after the surgery and maintaining the original outcome.

**Conclusions:**

External fixation using the J-EF could be considered as an alternative treatment for Mayo type II olecranon fractures as it appears to be a reliable, minimally invasive, and time-saving.

**Level of Evidence:**

Therapeutic Level IV.

## Introduction

Olecranon fractures are common injuries, accounting for approximately 20% of fractures of the proximal forearm in adults (1, 2). The mechanism of injury is usually a forceful contraction of the triceps during a fall on the outstretched hand, the humerus acting as a splitting wedge (3, 4). Although some patients can be managed nonoperatively, surgical fixation is usually necessary for displaced fractures (5, 6). Indications for surgical fixation include extensor mechanism weakness, intra-articular displacement, and instability of the humeroulnar joint (7, 8). The most commonly used surgical techniques for the treatment of olecranon fractures are tension-band wiring (TBW) fixation and plate fixation (PF). However, these techniques comes with some complications such as soft tissue injury due to the surgical exposure, postoperative discomfort of elbow due to prominence of metal work and the complications associated with removal of the hardware (9–11). To provide a simple, less invasive treatment option, we designed a J-shaped external fixator (J-EF) to treat Mayo type II olecranon fractures. With patent and license for clinical use, we applied the J-EF to treat 14 patients with Mayo type II olecranon fractures. The aim of this study is to introduce our experience with the use of the J-EF and report the long-term outcomes of patients.

## Materials and Methods

### Design and Structure of the J-Shaped External Fixator

**Figure 1:**
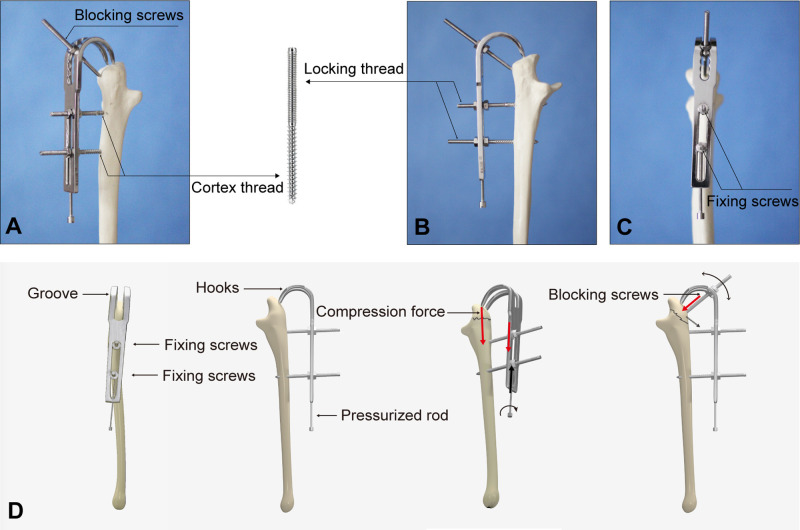
Structure of the J-shaped external fixator (J-EF). General view of the J-EF (**A**–**C**). Structure and the compression system of J-EF (**D**).

The design of J-EF was based on our previous anatomical study of olecranon from Asian adult cadavers for improved understanding of the anatomical structure of olecranon and for making our design compatible with the anatomical morphology of olecranon. The fixator was 4 × 120 × 14 mm in size, with a groove of 70 × 5 mm in the middle and two semielliptical hooks with 5 mm in between placed at the proximal part of the fixator, and the radian (*R*) of the hook was 15–20 mm. A longitudinal compression rod was equipped at the distal part of the fixator, which provided a longitudinal compression force and constituted the compression system together with the hooks and cortical screws (Figure 1A–C). The compression system works with the rotation of the pressurized rod in a clockwise manner to drive the fixator downward, therefore producing a compression force on the fracture site from the hooks (Figure 1D).

We designed the upper part of the fixing screw with the locking screw body, while the lower part with the cortical screw body; so as the screws could be firmly fixed to the body of the fixator through locking threads to achieve a rigid fixation to the proximal ulna from the cortical threads (Figure 1A–C). All the screws were 60 mm in length and 4 mm in diameter. The screws performed two functions in the current study: lower screws served as “fixing screws” to provide firm fixation of the fixator and proximal ulna, and the upper screws around the hook were “blocking screws” to prevent displacement of the fracture fragments (Figure 1D).

### Mechanical Performance Evaluation of the J-EF

By taking TBW as a control, biomechanical comparative studies were performed using six pairs of fresh-frozen cadaveric specimens with approval from the ethics committee of Hebei Medical University. The average age of specimens was 42.33 (range: 29–63) years. Fresh cadavers were divided into two groups (*n* = 6). The models of olecranon fractures were made in each group based on Mayo type II. The fractures were fixed using J-EF in the treatment group and TBW in the control group. The mechanical testing of the tensile strength was performed by a universal testing machine (Institute of Traumatology and Biomechanics, KEYI Med. Ltd., Beijing). The mechanical test was conducted in cyclic loading mode under 50–500 *N* loading at an amplitude of 225 *N* and frequency of 1 Hz. The test was run for 500 cycles, and the displacement of the fracture site was recorded by a reluctance transducer at 300 and 500 cycles, respectively. A static tensile strength test was performed with a loading speed of 10 mm/min, and fixation failure was defined as the displacement of the fracture site greater than 2 mm.

### Study Population

Between January 2002 and December 2005, we used the J-EF for the treatment of 14 patients with Mayo type II olecranon fracture. Table 1 lists the inclusion and exclusion criteria for the selection of patients. The mean operation time and intraoperative blood loss were compared with those of patients treated with TBW by the same group of doctors. Radiography was performed to assess the process of fracture healing. Improvement in range of motion, complications, and patient-reported outcome (Mayo Elbow Performance Score [MEPS] and Disabilities of the Arm, Shoulder, and Hand [DASH] scores) were recorded 6 months after surgery. Our study was conducted after obtaining the approval from the ethics committee and all patients were provided with informed consent before the surgery.

**Table 1:** Inclusion and exclusion criteria.

Inclusion criteria	Exclusion criteria
Age <75 years	Associated ligamentous injury
Mayo type II olecranon fractures	Stable structure failure
	(Mayo type III fractures including joint dislocation, A–P instability)
Fracture within 2 weeks	Associated vascular or nerve injury
Displaced fracture of the olecranon	Associated fractures of the coronoid, radius head, or distal aspect of the humerus
Minimal or moderate fragmentation of the olecranon	Dieses that are unable to follow rehabilitation training (Alzheimer's disease, etc.)

### Surgical Technique

Fixing screws were driven into the ulnar shaft to ensure the stability of the distal part during reduction. Closed reduction was then performed under fluoroscopy, with the assistance of Kirschner wires if needed. The elbow was gradually flexed to 90°, and two 5 mm incisions were made over the dorsal part of the olecranon process for insertion of the hooks of J-EF. The olecranon fixing screws were subsequently assembled with the fixator. Restoration of the articular surface was monitored radiologically as the fracture site was pressurized with the compression system of J-EF. The reduction was considered satisfactory if the step-off or gap of the articular surface was less than 1 mm.

### Postoperative Management and Outcome Assessment

Passive and active flexions (up to 90°) were initiated 2 days after the surgery. Unlimited flexion, pronation, and supination were initiated two weeks after surgery. Weight-bearing was not allowed until 2 months after surgery or until the removal of the external fixator. All patients were reviewed every two weeks after surgery. X-rays were obtained 8 weeks and 12 weeks postoperatively to confirm radiological healing. The J-EF was not removed until the fractures shown a gradual loss in fracture line on radiology. Infection, nonunion, deformity healing, postoperative pain, and elbow dysfunction were recorded as the adverse event. Clinical and functional evaluations were performed 6 months after surgery. Joint function was evaluated by the MEPS and DASH scores.

### Statistical Analysis

Continuous variables were summarized as means ± standard deviation, and categorical variables were summarized as absolute and relative frequency. Statistical analysis was performed using SPSS 22.0 (IBM Corp., Armonk, NY, USA). *P* < 0.05 was considered statistically significant.

## Results

### Structure of the J-Shaped External Fixator

The fixator was 4 × 120 × 14 mm in size, with a groove of 70 × 5 mm in the middle and two semielliptical hooks with 5 mm in between placed at the proximal part of the fixator, and the radian (*R*) of the hook was 15–20 mm. A longitudinal compression rod was equipped at the distal part of the fixator, which provided a longitudinal compression force and constituted the compression system together with the hooks and cortical screws (Figure 1A–C). The compression system works with the rotation of the pressurized rod in a clockwise manner to drive the fixator downward, therefore producing a compression force on the fracture site from the hooks (Figure 1D).

We designed the upper part of the fixing screw with the locking screw body while the lower part with the cortical screw body; thus, the screws could be firmly fixed to the body of the fixator through locking threads to achieve a rigid fixation to the proximal ulna from the cortical threads (Figure 1A–C). All the screws were 60 mm in length and 4 mm in diameter. The screws performed two functions in the current study: lower screws served as “fixing screws” to provide firm fixation of the fixator and proximal ulna, and the upper screws around the hook were “blocking screws” to prevent displacement of the fracture fragments (Figure 1D).

### Biomechanical Properties of J-EF

**Figure 2:**
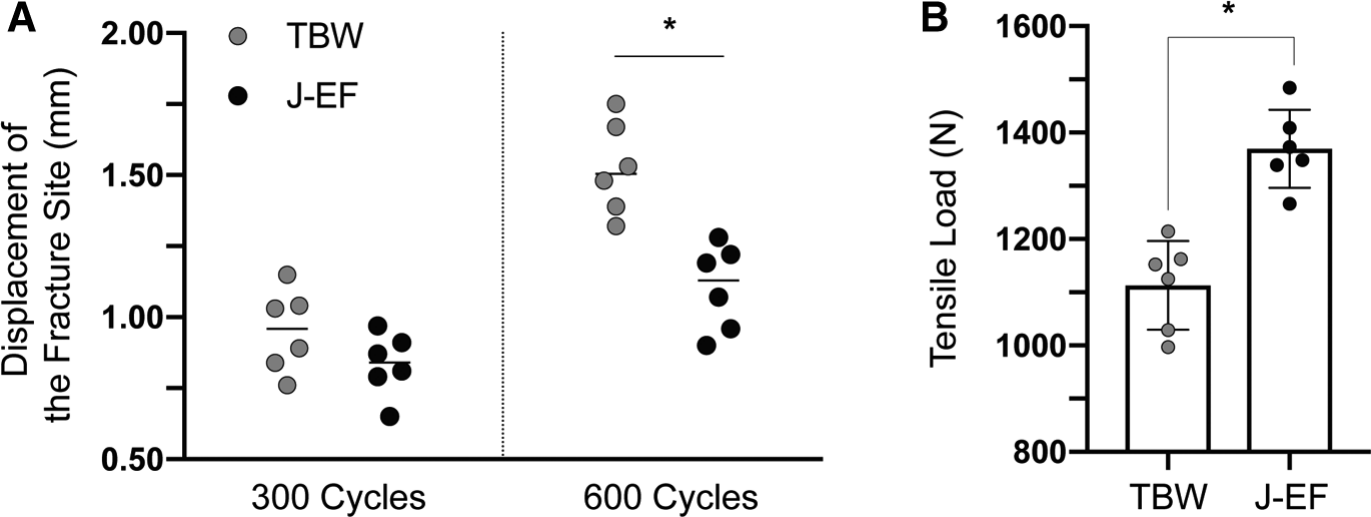
Biomechanical properties of J-EF. Tensile strength of J-EF was tested using tension-band wiring (TBW) as the control. The tensile strength of the J-EF group was greater than that of the TBW group after running 600 cycles (*P* < 0.05). Similar results were found in the static tensile loading test between TBW and J-EF. The difference between the two groups was analyzed by Student’s *t*-test.

Shown as the comparative biomechanical data in Figure 2, the tensile strength of the J-EF group with a mean displacement of 1.1 mm (0.15 mm; range: 0.9–1.28 mm) was greater than that of the TBW group with a mean displacement of 1.52 mm (0.16 mm; range: 1.32–1.75 mm) after running 600 cycles (*P* < 0.05). Similar significant differences (*t* = −5.666, *P* = 0) were found in the static tensile loading test between TBW and J-EF, which was 1,113.17 *N* (83.4 *N*; range: 997–1,214 *N*) and 1,369.83 *N* (73.19 *N*; range: 1,266–1,484 *N*), respectively. The difference between the two groups was analyzed by Student’s *t*-test. The comparative results indicate the noninferiority of J-EF to TBW in biomechanical properties and provide a biomechanical precondition for the clinical application of J-EF.

### Patients

The 14 patients (nine males; 64.3%) recruited for our study had a mean age of 47 ± 14.7 (range: 25–67) years. The fracture was on the left side in 11 (78.6%) patients (Table 2). Four (28.6%) patients had Mayo IIB fractures, and ten (71.4%) had Mayo IIA fractures. The most frequent mechanism of injury was falling from height (*n* = 8, 57.1%), followed by sports injury (*n* = 3, 21.4%) and traffic accident (*n* = 2, 14.3%); the remaining patient is a swimmer and injured during training practice. Nine (64.3%) patients had documented comorbidities. The American Society of Anesthesiologists (ASA) grade was not significantly different between male and female patients (*P* = 0.126).

**Table 2:** Patient demographics and outcomes.

	Male (*n *= 9)	Female (*n *= 5)	*P*-value
Age (year)^a^	41.6 (13.7, 25–64)	56.8 (10.9, 38–67)	0.071^d^
Age (year)^b^	36 (31, 54)	63 (51, 65)	
Mechanism of injury^c^			0.147^e^
Fall from height	3 (21.4%)	5 (35.7%)	
Sports injury	3 (21.4%)	0 (0%)	
Motor-vehicle collision	2 (14.3%)	0 (0%)	
Other	1 (7.1%)	0 (0%)	
ASA grade^c^			0.126^e^
1	5 (35.7%)	0 (0%)	
2	3 (21.4%)	4 (28.6%)	
3	1 (7.1%)	1(7.1%)	
Range of motion^a,b^			
Flexion arc (deg)	130.6 (9, 115–145)	136 (8.6, 125–150)	0.326^d^
Rotation arc (deg)	174.4 (5, 170–180)	172 (7.5, 160–180)	0.699^f^
MEPS^a,b^	94.4 (8.3, 80–100)	93 (7.5, 80–100)	0.606^f^
DASH score^a,b^	15.5 (6.6, 5–23.3)	11.7 (7.9, 3.3–24.2)	0.326^d^

^a^ *Values are presented as mean* (*SD, range*)*.*

^b^ *Values are presented as median* (*Q1, Q3*)*.*

^c^ *Values are presented as the number of patients* (*percent*).

^d^ *Student’s t-test.*

^e^ *Fisher’s exact test.*

^f^ *Mann–Whitney U test.*

*MEPS, Mayo Elbow Performance Score; DASH, Disabilities of the Arm, Shoulder, and Hand*.

### Operative Time and Intraoperative Blood Loss

**Figure 3:**
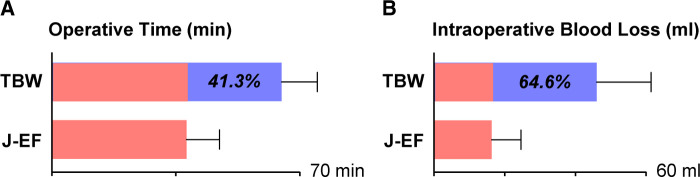
Comparative study of perioperative clinical data between TBW and J-EF treatment. A comparative study of perioperative data including operative time (**A**) and intraoperative blood loss (**B**) between J-EF and TBW was performed according to the anesthesia sheets and suction device; the percentage represents the reduction in the J-EF group relative to the TBW group.

For better evaluation of the clinical application of J-EF, we performed statistical analysis on the perioperative clinical data, including operative time and intraoperative blood loss; data were compared with TBW treatment. As shown in Figure 3, the mean operative time was 38 ± 9.6 (range: 23–62) min, which was lower than the mean operative time in a comparable group of patients treated by TBW at our hospital during the same period (64.7 ± 10.4 [range: 48–85] min); the difference was statistically significant (*t* = 6.79, *P* < 0.001). Similarly, the mean intraoperative blood loss of the J-EF group was 16.4 ml (range: 5–35 ml; SD: 8.5 ml), which was significantly less (*t* = 6.01, *P* < 0.001) than that of the TBW group (mean: 46.4 ml; range: 30–80 ml; SD: 15.9 ml). For accurate measurements, the intraoperative blood loss was calculated according to the suction device; therefore, the true volume of blood loss was less than this amount. According to our experience, intraoperative blood loss using J-EF should be less than 5 ml; therefore, tourniquets are not necessary when using J-EF treatment for most cases. With less blood loss and shorter operation time, J-EF makes the treatment of olecranon fractures more in line with the requirements of ambulatory surgery.

### Clinical Outcome and Long-Term Review

**Figure 4:**
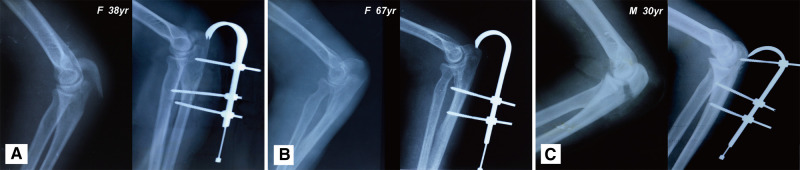
Preoperative and postoperative X-rays of the elbow joint. Typical cases treatment with J-EF in our study. (**A**) Female patient of 38 years, car accident; (**B**) female patient of 67 years, fall from height; and (**C**) male patient of 30 years, sports injury.

At 6 months after surgery, the mean elbow flexion arc of patients treated with the J-EF was 132.5° ± 9.2° (range: 115°–150°) and the mean forearm rotation arc was 173.6° ± 6.1° (range: 160°–180°). The mean DASH score was 14.1 ± 7.3 (range: 3.3–24.2) points, and the mean MEPS was 94 ± 8.1 (range: 80–100) points (Table 2). Range of motion and the MEPS and DASH scores were not significantly different between male and female patients (all *P* > 0.05; Table 2). The mean time to removal of J-EF was 67.1 ± 13.2 (range: 45–97) days. One patient complained of mild intermittent pain in the elbow 2 weeks after surgery, but the pain was relieved after the removal of the fixator with the healing of the fracture. No patient had significant complications including nonunion, malunion, and infection during our 1-year follow-up after surgery. Preoperative and postoperative X-rays of our typical cases are shown in Figure 4.

We reviewed and followed up our first group of patients underwent J-EF treatment, who were more than 15 years after surgery, X-rays and the follow-up of typical case was shown in Supplemental Figure 1. Unfortunately, among the first group of 14 patients, we could only be able to follow-up eight patients; the other six patients were lost due to death of natural causes. With a follow-up of the eight patients, all patients achieved a good elbow joint function score, and no complaint of pain or limited range of motion of the elbow was noticed (Table 3).

**Table 3:** Long-term review of patients more than 15 years postoperation.

Case No.		Age (year)	Gender	Post OP (year)	Range of motion (deg)		Joint function	
					Flexion	Rotation	MEPS	DASH
009706	***Yong***	47	M	16	135	170	100	0.83
015419	***Xue.p***	46	M	16	145	180	100	3.3
019732	***Xiu.z***	40	M	15	145	180	100	0
042356	***Guo***	51	M	15	125	180	100	10.8
031187	***Jian.h***	69	M	15	130	170	90	5
051442	***Cui***	80	F	15	125	160	95	13.3
038324	***Hui.p***	67	F	16	140	180	100	0
077845	***Shu.m***	78	F	15	135	170	85	10

*MEPS, Mayo Elbow Performance Score; DASH, Disabilities of the Arm, Shoulder, and Hand.*

## Discussion

For olecranon fracture, which accounts for approximately 8%–10% of all elbow fractures, the surgical goals are to restore articular integrality, provide stable, reliable fixation and minimize joint stiffness via early mobilization. Typically, most displaced olecranon fractures are managed with open reduction and fixation. TBW is a commonly used surgical fixation technique (12); the prominence of metalwork and soft tissue complications are major concerns and the rates of TBW removal are high (13–16), the external fixator that we used was designed to avoid these problems. The mechanical study has shown that the tensile strength of J-EF is reliable and not inferior to that of TBW. The efficacy of J-EF is attributable to the firm hold of the fracture site achieved via hooks and screws. The fracture is also stabilized by the compression force. Meanwhile, TBW acts like an indirect strapping of the fracture because of the coverage by fascial tissue and the triceps brachii tendon. Due to the elastic mechanical property of TBW, there is a possibility of loosening during postoperative joint activities. Thus, J-EF provides a more reliable fixation for the fracture. Furthermore, we have found that compression adjustment is easily performed through the pressurized rod within 48 h post-surgery if postoperative radiography does not show a satisfactory reduction.

C-arm fluoroscopy was used to ensure anatomical reduction. Rehab activities including gravity-assisted elbow flexion exercises could be initiated within 48 h after surgery because of the absence of plaster immobilization. The time to remove the fixator was 45–97 days. All our cases met the standard of clinical healing with no reports of nonunion, delayed healing, or refracture during the follow-up period. The minimally invasive reduction—with preservation of the periosteum and the subdermal vascular network—can be especially advantageous for professional athletes (12). By way of example, one high-quality athlete in our study, a 13-year-old male diver, underwent J-EF fixation. Elbow function recovered without malunion in 6 months, and no symptoms of traumatic arthritis were found during the long-term follow-up. Besides the case series represented in our study, we also treated a small number of Mayo type IIIa fractures with J-EF fixation and achieved good results. Although open reduction fixation is not the purpose of designing J-EF, minimal incision at the fracture site will be helpful and necessary for the reduction of Mayo type IIIa fractures, according to our experience. However, it must be noted that this technique may not be applicable to highly unstable fractures (for example, Mayo type IIIb); for such patients, we still recommend open reduction and plate fixation (15, 17). Due to limitations on the number of cases, we did not find a significant difference in clinical outcomes of using J-EF between Mayo type IIa and IIb fractures in our present study. Hopefully, we could perform a comparative study on the treatment outcome of J-EF treatment between different types of fractures in our further study. With a relatively small number of included cases, however, this study is limited by the need for sufficient patients to support the feasibility of the study. We are also trying to carry out the dynamic biomechanical study of J-EF after implantation using medical computer technology (18). If possible, we will also use medical imaging and computer technology to conduct a surgical simulation of J-EF treatment for olecranon fractures.

## Conclusions

To sum up, being simple to use, less invasive, and providing early rehabilitation, the J-EF may become a reliable treatment option for Mayo type II olecranon fractures.

## Data Availability

The original contributions presented in the study are included in the article/Supplementary Material; further inquiries can be directed to the corresponding author/s.

## Supplementary Material

**Supplementary Figure 1** | Typical case of Mayo type IIa olecranon fracture treated with J-EF fixation. Male patient, 30 years, traffic accident. Preoperative (**A**) and postoperative X-rays of the elbow joint were shown in **A-D**. The elbow joint function 3 months postsurgery (**E-G**) as well as 16 years follow-up (**H-J**) of the patient were shown in the supplementary data.
