# Supplementary figures and images for: Case Report: J-Shaped External Fixator for Treatment of Mayo Type II Olecranon Fractures – A Novel Surgical Technique and Report of Clinical Applications
Source: Front Surg. 2022 Jun 15;9:855600. doi: 10.3389/fsurg.2022.855600 (PMC9240390; doi:10.3389/fsurg.2022.855600)

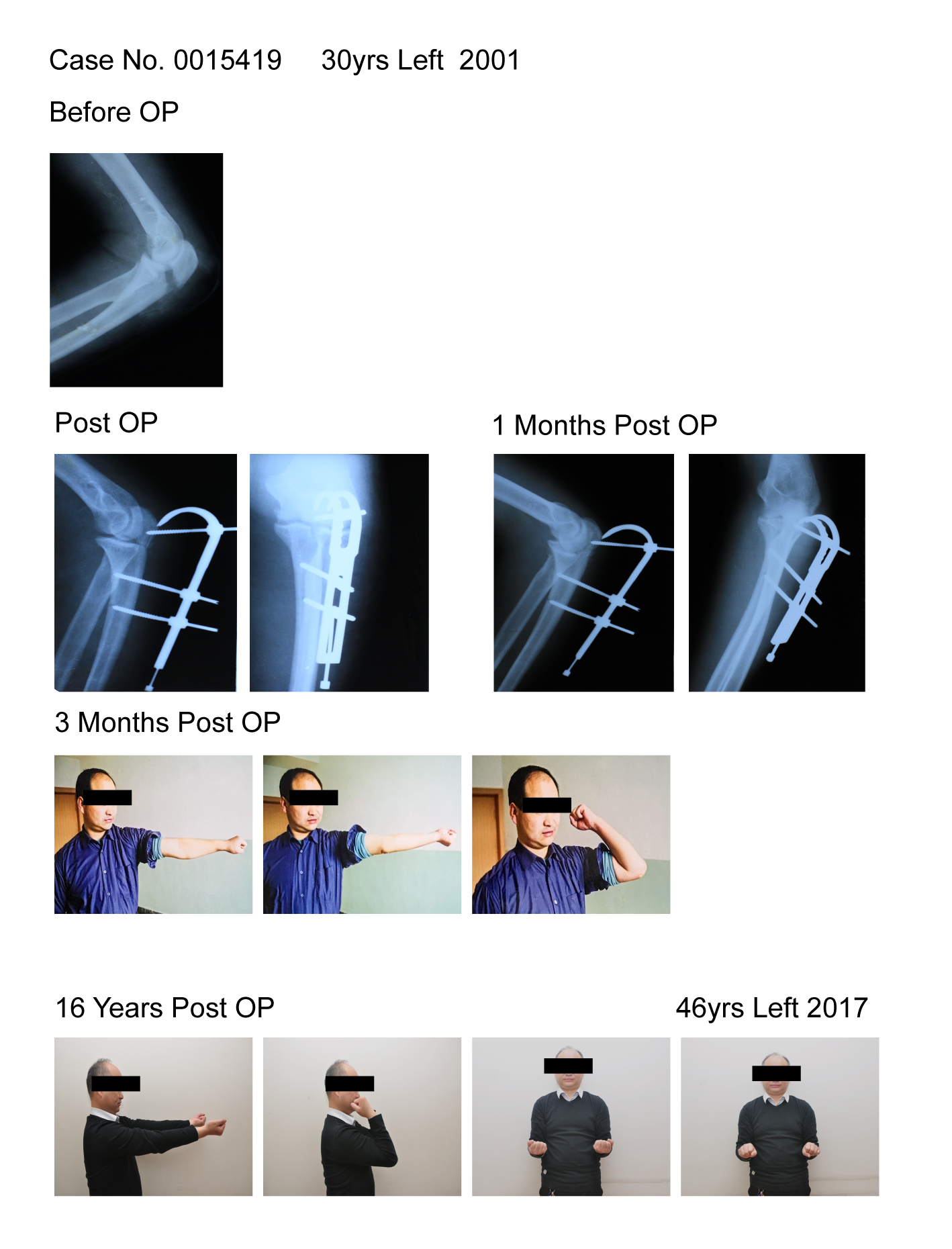

Supplement: Supplementary file 1 [file Image_1_v1.tif]
